# Cryopreservation of Neurospheres Derived from Human Glioblastoma Multiforme

**DOI:** 10.1634/stemcells.2008-0009

**Published:** 2009-01

**Authors:** Yuk-Kien Chong, Tan-Boon Toh, Norazean Zaiden, Anuradha Poonepalli, Siew Hong Leong, Catherine Ee Ling Ong, Yiting Yu, Patrick B Tan, Siew-Ju See, Wai-Hoe Ng, Ivan Ng, Manoor P Hande, Oi Lian Kon, Beng-Ti Ang, Carol Tang

**Affiliations:** a Singapore Institute for Clinical Sciences, Genome Institute of Singapore, Agency for Science, Technology and ResearchSingapore; b Department of Research, National Neuroscience InstituteSingapore; c Department of Physiology, Yong Loo Lin School of Medicine, National University of SingaporeSingapore; d Division of Medical Sciences, Humphrey Oei Institute of Cancer Research, National Cancer CentreSingapore; e DSO National Laboratories (Kent Ridge)Singapore; f Cell and Medical Biology, Genome Institute of Singapore, Agency for Science, Technology and ResearchSingapore; g Duke-National University of Singapore Graduate Medical SchoolSingapore; h Department of Neurology, National Neuroscience InstituteSingapore; i Department of Neurosurgery, National Neuroscience InstituteSingapore

**Keywords:** Glioma, Neurosphere, Vitrification, Cryopreservation, Cancer stem cell

## Abstract

Cancer stem cells have been shown to initiate and sustain tumor growth. In many instances, clinical material is limited, compounded by a lack of methods to preserve such cells at convenient time points. Although brain tumor-initiating cells grown in a spheroid manner have been shown to maintain their integrity through serial transplantation in immune-compromised animals, practically, it is not always possible to have access to animals of suitable ages to continuously maintain these cells. We therefore explored vitrification as a cryopreservation technique for brain tumor-initiating cells. Tumor neurospheres were derived from five patients with glioblastoma multiforme (GBM). Cryopreservation in 90% serum and 10% dimethyl sulfoxide yielded greatest viability and could be explored in future studies. Vitrification yielded cells that maintained self-renewal and multipotentiality properties. Karyotypic analyses confirmed the presence of GBM hallmarks. Upon implantation into NOD/SCID mice, our vitrified cells reformed glioma masses that could be serially transplanted. Transcriptome analysis showed that the vitrified and nonvitrified samples in either the stem-like or differentiated states clustered together, providing evidence that vitrification does not change the genotype of frozen cells. Upon induction of differentiation, the transcriptomes of vitrified cells associated with the original primary tumors, indicating that tumor stem-like cells are a genetically distinct population from the differentiated mass, underscoring the importance of working with the relevant tumor-initiating population. Our results demonstrate that vitrification of brain tumor-initiating cells preserves the biological phenotype and genetic profiles of the cells. This should facilitate the establishment of a repository of tumor-initiating cells for subsequent experimental designs.

## INTRODUCTION

Cancers have long been regarded as morphologically heterogeneous masses of cells [1]. Cancer stem cells have been shown to be responsible for tumor initiation and maintenance in many neoplasms of the hematopoietic system, breast, brain, prostate, colon, head and neck, and pancreas [2–10]. They have been shown to possess chemoresistance and radioresistance properties [11–13], thus making them plausible candidates for the perpetuation or recurrence of tumor growth following treatment regimens. In many studies involving the prospective isolation of tumor-initiating cells, only small amounts of clinical material are available, and this limitation is compounded by a lack of methods to preserve such cells at convenient time points. In brain tumors, for instance, it has been demonstrated that in vivo serial passage of tumor neurospheres (a heterogeneous mix of stem and progenitor cells) can provide a means to reliably maintain such primary cell lines [14]; however, in practice it is not always possible to have access to immune-compromised animals of suitable ages to continuously maintain the stem and progenitor cells. Lee et al. showed that tumor stem-like cells grown in serum-free conditions closely mimic the genotype, gene expression profile, and biology of their parental tumors [15]. These observations bring into question the relevance of standard serum-grown cancer cell lines for studying the biology of human cancer and for screening new therapeutic agents. We therefore sought to explore a novel method of vitrification for brain tumor neurospheres that is effective at preserving the cells' biological and genetic properties. We believe the method could provide many researchers with the means to establish a repository of primary brain tumor-initiating cells that can readily be tapped upon for expansion or experimental designs.

Vitrification has been commonly used in the preservation of cells involved in reproduction [16,17], human embryonic stem cell bodies [18], and cell-containing constructs used in tissue engineering [19]. Vitrification is preferred over conventional slow-cooling methods for several reasons (reviewed in [20]), with one central dogma: when thawed, cells should remain viable and maintain their biological profiles. Here, we report the development of a modified vitrification technique applied to long-term cryopreservation of brain tumor neurospheres derived from human adult malignant glioblastoma multiforme (GBM). We propose a method whereby vitrification can be combined with in vivo serial passaging to establish a repository of tumor-initiating cells that should facilitate future investigative work.

## MATERIALS AND METHODS

### Tissue Collection and Primary Neurosphere Culture

Graded brain tumor specimens were obtained with informed consent, as part of a study protocol approved by the institutional review board. In this study, S0305 was from a patient with recurrent GBM (grade IV) who had received radiation therapy, and S0405, S0805, S0306, and S0807 were from patients with primary GBM who were treatment-naive. Tumors were processed according to Gritti et al. [21] with slight modifications. Cells were seeded at a density of 2,500 cells per cm^2^ in chemically defined serum-free selection growth medium consisting of basic fibroblast growth factor, basic fibroblast growth factor (bFGF; 20 ng/ml; Chemicon, Temecula, CA, http://www.chemicon.com), epidermal growth factor (EGF; 20 ng/ml; Chemicon), human recombinant leukemia inhibitory factor (LIF; 20 ng/ml; Chemicon), heparin (5 μg/ml; Sigma-Aldrich, St. Louis, http://www.sigmaaldrich.com), and serum-free supplement (B27; 1×; Gibco, Grand Island, NY, http://www.invitrogen.com) in a 3:1 mix of Dulbecco's modified Eagle's medium (DMEM; Sigma-Aldrich) and Ham's F-12 Nutrient Mixture (F12; Gibco). The cultures were incubated at 37°C in a water-saturated atmosphere containing 5% CO_2_ and 95% air. To maintain the undifferentiated state of neurosphere cultures, growth factors were replenished every 2 days. Differentiation was carried out over 14 days in DMEM/F12 without growth factors, supplemented with 5% fetal bovine serum (FBS) and B27. Successful neurosphere cultures (1–4 weeks) were expanded by mechanical trituration using a flame-drawn glass Pasteur pipette, and cells were reseeded at 100,000 cells per milliliter in fresh medium.

### Cryopreservation and Thawing of Neurosphere Cultures for Viability Count

In the conventional cryopreservation method, 5,000 neurospheres (50–100 μm) were frozen in a slow-cooling protocol using a freezing container (Mr. Frosty; Nalgene Nunc International, Rochester, NY, http://www.nalgenelabware.com) in −80°C for 24 hours before transfer into −196°C liquid nitrogen storage for 30 days. Freezing media used contained DMEM/F12 supplemented with 10% dimethyl sulfoxide (DMSO; Merck & Co., Whitehouse Station, NY, http://www.merck.com) only, or 10% DMSO and 90% FBS (HyClone, Logan, UT, http://www.hyclone.com). These samples were thawed at 37°C in a water bath for 1–2 minutes and washed with excess DMEM/F12 before being cultured in chemically defined serum-free selection growth medium supplemented with growth factors (DMEM/F12; 20 ng/ml each of EGF, bFGF, and LIF; B27; and 5 μg/ml heparin). Viability counts were carried out after incubation periods of 1, 5, and 10 days [22].

In the vitrification method, tumor neurospheres from the same passage were subjected to either vitrification or continuous culturing (nonvitrified). Five thousand neurospheres (50–100 μm) were frozen in a rapid cooling protocol. Neurospheres were resuspended in a holding medium (HM) of DMEM/F12 containing HEPES buffer (Gibco) with or without 20% FBS before being transferred by pipetting into sequentially increasing concentrated vitrification solutions (VS1 and VS2). Neurospheres were incubated for 1 minute in VS1 consisting of 10% DMSO and 10% ethylene glycol (EG) (Merck), followed by a 25-second incubation in VS2 consisting of 20% DMSO, 20% EG, and 0.3 M sucrose. The mixture was immediately transferred into 0.78-mm inner diameter borosilicate glass capillaries (Harvard Apparatus, Holliston, MA, http://www.harvardapparatus.com), snap-frozen, and stored in liquid nitrogen. All procedures were performed in an aseptic manner at room temperature.

The following periods of freezing for vitrified cultures were evaluated prior to thawing: S0305 and S0405 for 30 days, S0807 for 8 months, S0306 for 1.5 years, and S0805 for 2.5 years. Thawing was performed in sucrose solutions (SS) of sequentially decreasing concentrations (SS1 and SS2). After removal from liquid nitrogen, the contents of the glass capillaries were released by reverse capillary action into SS1 containing HM supplemented with 0.2 M sucrose for 1 minute. They were then transferred by pipetting into SS2 containing HM supplemented with 0.1 M sucrose and incubated for 5 minutes, followed by another 5 minutes in HM alone. The mixture was washed with excess HM before being cultured in chemically defined serum-free selection growth medium supplemented with growth factors at the above-mentioned concentrations (DMEM/F12, EGF, bFGF, LIF, B27, and heparin). Viability counts were carried out after incubation periods of 1, 5, and 10 days [22].

### Immunofluorescence Analyses

Neurospheres from vitrified and nonvitrified conditions were dissociated into single cells using Accutase (eBioscience Inc., San Diego, http://www.ebioscience.com; non-trypsin-based) and seeded at a cell density of 2 × 10^5^ cells per well of laminin-coated (Sigma-Aldrich) eight-well culture slides (BD Falcon, Bedford, MA, http://www.bdbiosciences.com). Plated cells were then stained for the following markers.

#### Stemness Markers

The undifferentiated cells (stem state) were stained for Nestin (Chemicon), Oct-4 (Santa Cruz Biotechnology Inc., Santa Cruz, CA, http://www.scbt.com), Musashi-1 (Chemicon), and Ki-67 (Chemicon). Incubation with a secondary antibody conjugated to Alexa-Fluor (Molecular Probes, Eugene, OR, http://probes.invitrogen.com) was carried out.

#### Multipotentiality Markers

Induction of differentiation was carried out with DMEM/F12 in the absence of growth factors and supplemented with 5% FBS and 1× B27. On day 14, differentiated cells were stained for neurons (neuron-specific class III beta-tubulin [TuJ1]; Chemicon), astrocytes (glial fibrillary acidic protein [GFAP]; Dako, Glostrup, Denmark, http://www.dako.com), and oligodendrocytes (O4; Chemicon).

### Secondary Sphere Formation Assays

Tumor neurospheres were dissociated into single cells by treatment with Accutase (eBioscience). The cells were then dispensed into each well of a 96-well plate at decreasing cell numbers of 100, 80, 60, 40, and 20. Sphere formation was scored at day 7 after seeding. To carry out sequential minimal dilution assays, the secondary spheres were similarly dissociated into single cells and then dispensed into each well of a 96-well plate at similar decreasing numbers. Tertiary sphere formation was scored on day 7 after seeding. Sequential minimal dilution experiments were carried out for at least three passages.

### Flow Cytometry

Neurospheres were dissociated with Accutase (eBioscience) and stained with anti-CD133/2-allophycocyanin according to the manufacturer's instructions (Miltenyi Biotec, Bergisch Gladbach, Germany, http://www.miltenyibiotec.com). Dead cells were distinguished by propidium iodide staining. A total of 10,000 events was acquired on a FACSCalibur instrument (BD Biosciences, San Diego, http://www.bdbiosciences.com). Data were plotted using FlowJo software (Tree Star, Ashland, OR, http://www.treestar.com).

### Karyotypic Analyses of Tumor Neurospheres

Five thousand cells from triturated neurospheres were cultured in the stem state on a laminin-coated culture-well slide. The cells were then treated within 3–5 days with 0.1 μg/ml colcemid (Invitrogen, Carlsbad, CA, http://www.invitrogen.com) for 24 hours, followed by 0.075 M KCl, and fixed in methanol:acetic acid (3:1). Metaphase-fluorescent in situ hybridization (mFISH) (MetaSystems XCyte mFISH; MetaSystems GmbH, Heidelberg, Germany, http://www.metasystems.de) and spectral karyotyping (SkyPaint; Applied Spectral Imaging, Israel, http://www.spectral-imaging.com) were performed on metaphases according to the manufacturers' instructions.

### Microarray Analysis and Quantitative Real-Time Polymerase Chain Reaction

Total RNA was isolated from neurosphere cells using Trizol (Invitrogen) and purified with the RNeasy Mini Kit (Qiagen, Hilden, Germany, http://www1.qiagen.com). Reverse transcription and cRNA amplification were performed using an RNA Amplification kit (Ambion, Austin, TX, http://www.ambion.com). The microarray hybridization was performed using the Illumina Gene Expression BeadChip (Illumina Inc., San Diego, http://www.illumina.com), and data analysis was performed using GeneSpring software (Agilent Technologies, Palo Alto, CA, http://www.agilent.com).

Real-time polymerase chain reaction (PCR) was carried out according to the manufacturer's protocol for the LightCycler FastStart MasterPLUS SYBR Green I real-time PCR kit (Roche Diagnostics, Basel, Switzerland, http://www.roche-applied-science.com). A standardized amount of 50 ng of cDNA was used for each PCR. The PCR was carried out with specific oligonucleotide primer pairs at the optimized annealing temperatures stated (supporting information Table 1). Cycle parameters on the LightCycler (Roche Diagnostics) were 40 cycles of 95°C for 10 seconds, 55°C for 10 seconds, and 72°C for 5 seconds. Each real-time PCR was done in triplicate, and the level of expression of each gene was determined relative to the normalizer gene glyceraldehyde-3-phosphate dehydrogenase.

### Stereotaxic Intracranial Implantations of NOD/SCID Mice

Tumorigenicity was determined by injecting GBM cells from dissociated neurospheres orthotopically in nonobese diabetic/severe combined immunodeficiency (NOD/SCID) mice. Two hundred thousand cells in 5 μl of phosphate-buffered saline were delivered into the right frontal lobe (0.5 μl/minute) by stereotaxic injection through a glass electrode connected to a Hamilton syringe (Narishige, Tokyo, http://www.narishige.co.jp/main.htm). The following coordinates were used: anteroposterior, +2 mm; mediolateral, +2 mm; dorsoventral, −3 mm. Mice were euthanized by means of transcardiac perfusion with 4% paraformaldehyde upon presentation of neurological deficits with ataxia, cachexia, lethargy, or seizure [23]. Hematoxylin and eosin staining and immunohistochemistry were performed on 5-μm-thick paraffin sections. Sections were processed as described by Vescovi et al. [24]. Mouse anti-human vimentin antibody staining (clone V9; Zymed Laboratories, San Francisco, http://www.invitrogen.com) was used to confirm the presence of engrafted human cells.

### Statistical Analysis

Data are expressed as means ± SEM of at least three independent experiments. Student's *t* test or the Mann-Whitney *U* test was used where appropriate. *p* < .05 was accepted as statistically significant.

## RESULTS

### Vitrification Maintains the Morphology and Viability of Progenitor-Like Cells

To assess the effectiveness of vitrification over conventional slow-cooling methods, we analyzed essential properties, such as viability, expression of stem cell markers, and multipotentiality. All patients' lines generated free-floating neurospheres except for S0306, which generated semiadherent spheres. GBM neurospheres were frozen either conventionally in a slow-cooling protocol with 10% DMSO in the presence or absence of 90% FBS, or vitrified in low serum-containing or serum-free medium by exposing glass capillaries containing neurosphere clumps to liquid nitrogen. The cell clumps were then stored in liquid nitrogen for 30 days to as long as 2.5 years to mimic long-term storage prior to analyses.

**Figure 1:**
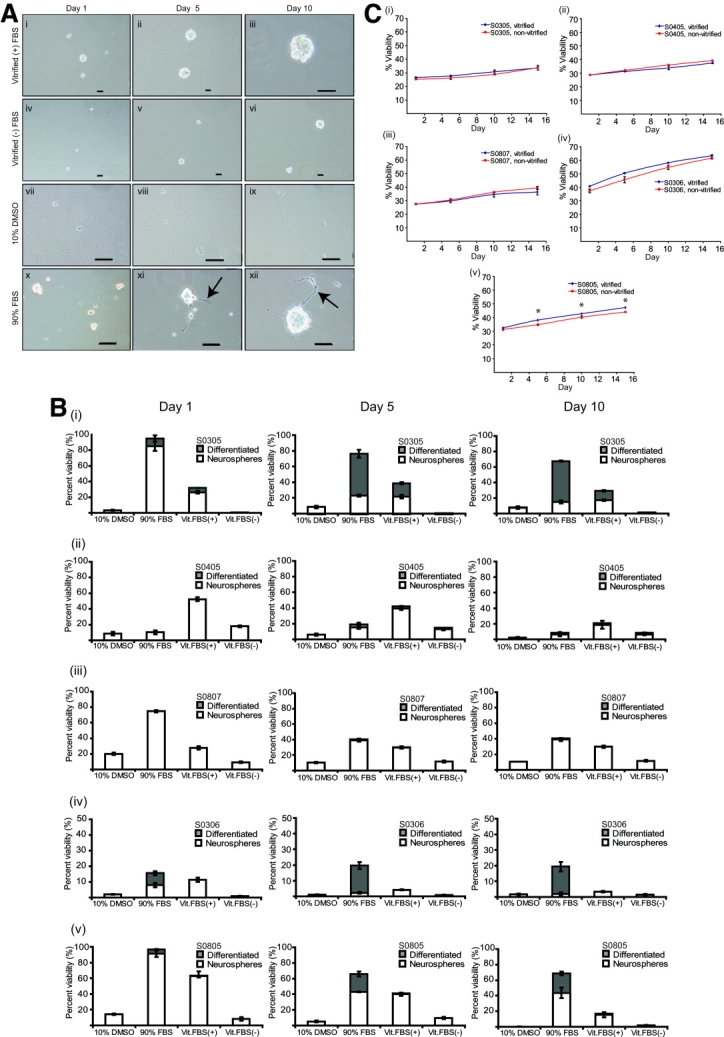
Vitrification results in greater viability and maintains proliferative capacity of tumor neurospheres. Tumor neurospheres were frozen by various methods: vitrification with 20% serum **(Ai–Aiii)**, vitrification without serum **(Aiv–Avi)**, 10% DMSO **(Avii–Aix)**, and 90% FBS **(Ax–Axii)**. After storage in liquid nitrogen for 30 days (vitrification with and without serum, 10% DMSO, and 90% FBS) and up to 2.5 years (vitrification with serum only), the neurospheres were thawed and subjected to morphological analyses while in culture under serum-free conditions supplemented with growth factors. Shown are representative images obtained from one patient's neurosphere line, S0305. Note the appearance of extended processes at the periphery (typical of differentiation) on days 5 and 10 of the sample frozen with 90% FBS (arrows). Scale bars = 100 μm. **(B):** Morphological analyses in **(A)** were quantified. **(A, B):** Experiments carried out in duplicate. **(C):** Patients' tumor neurospheres were subjected to vitrification, and proliferation rate using a standard AlamarBlue assay (AbD Serotec, Oxford, United Kingdom, http://www.ab-direct.com) was determined after thawing. S prefix represents tumor neurospheres, and numbers represent codes assigned to specimens (∗, *p* < .05). Abbreviations: DMSO, dimethyl sulfoxide; FBS, fetal bovine serum; Vit. FBS, vitrification with fetal bovine serum.

We assessed the viability of tumor neurospheres at 1, 5, and 10 days after thawing from liquid nitrogen storage by counting the number of neurospheres measuring at least 50–100 μm in diameter [22]. Neurosphere formation had previously been shown to indicate viability and proliferation [25,26]. A visual scan of cellular morphology indicated that vitrification with low serum best maintains initial frozen neurosphere size with little or no cell death, with cells remaining relatively undifferentiated for up to 15 days in culture (Fig. 1Ai–1Aiii, 1B). Cryopreservation by vitrification lacking serum or by standard freezing with 10% DMSO showed greater cell death and vastly smaller neurospheres compared with nonvitrified cultures, suggesting disintegration of sphere structures (Fig. 1Aiv–1Aix). We could not recover sufficient cells for further analysis due to extensive cell death. Standard freezing with 90% FBS yielded the best viability and preservation of sphere structures for all samples except S0405, where vitrification with serum yielded the best viability (Fig. 1Bii). However, the peripheries of all tumor spheres cryopreserved in 90% FBS exhibited clear signs of differentiation by 5 and 10 days post-thawing (Fig. 1Axi, 1Axii, arrows; Fig. 1B). Our finding indicates that freezing with 90% serum and 10% DMSO is an attractive alternative that should be explored in future studies. Encouraged by the good viability and lack of differentiation demonstrated by vitrified tumor spheres, we proceeded with our analyses by comparing vitrified and nonvitrified samples.

### Vitrification Maintains the Proliferation Rate of Tumor Neurospheres

A key criterion for efficacious vitrification is the preservation of cellular properties upon thawing when compared with their corresponding nonvitrified samples. Proliferation assays showed that all vitrified and nonvitrified tumor neurospheres continued to proliferate at similar rates except for S0805, which displayed a moderate but significant change (Fig. 1C).

### Vitrification Preserves the Stemness Expression and Multipotentiality of Tumor Neurospheres

**Figure 2:**
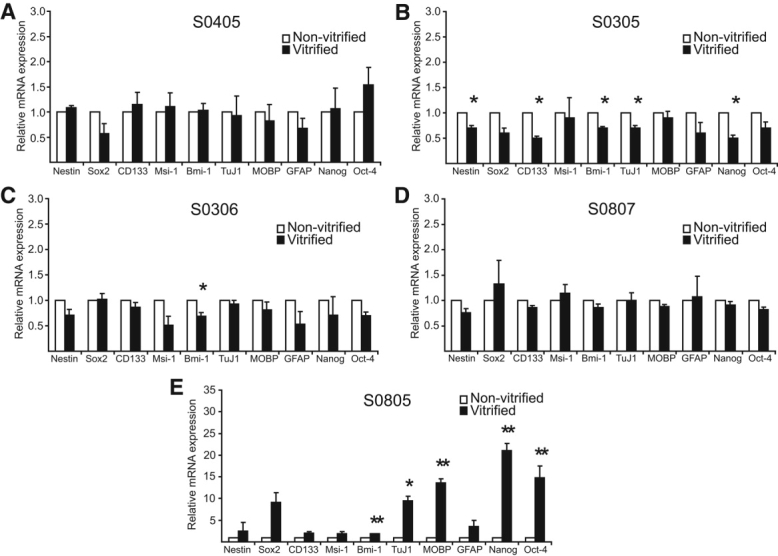
Vitrification preserves essential neural precursor gene expression. **(A–E):** Quantitative real-time polymerase chain reaction analysis of five patients' tumor neurospheres. The undifferentiated states of both vitrified and nonvitrified neurospheres were analyzed for the presence of stem/progenitor and differentiation markers (∗, *p* < .05; ∗∗, *p* < .01). Abbreviations: GFAP, glial fibrillary acidic protein; MOBP, myelin-associated oligodendrocyte basic protein; Msi-1, Musashi-1; TuJ1, neuron-specific class III beta-tubulin.

Markers of the stemness state, such as Nestin, Sox-2, CD133, Musashi-1 (Msi-1), Bmi-1, Nanog, and Oct-4, were assayed by quantitative real-time PCR. Differentiation markers, such as TuJ1, myelin-associated oligodendrocyte basic protein (MOBP), and GFAP, were also evaluated, as neurospheres are heterogeneous and comprise more differentiated progenitors, in addition to stem cells [27,28]. Nestin is expressed in neural precursors [29]; *Sox-2* is a gene known to play a role in maintenance of the neural progenitor state [30]; CD133 is a marker for neural stem cells, as well as brain tumor stem cells [10,31]; Msi-1 is a marker for self-renewal [32]; *Bmi-1* is a Polycomb group gene and epigenetic silencer that prevents premature growth arrest in most differentiated tissue cells and is essential for the self-renewal of several types of adult stem cells [33,34]; Nanog is a transcription factor essential for the maintenance of an undifferentiated state [35]; and Oct-4 is a transcription factor implicated in maintaining the pluripotency of stem cells [36,37]. TuJ1 marks neurons, MOBP marks oligodendrocytes, and GFAP marks astrocytes. Vitrification preserved the expression of essential stem cell markers for samples S0405, S0306, and S0807 (Fig. 2). Between vitrified and nonvitrified samples, expression of Nestin, CD133, Bmi-1, Nanog, and TuJ1 for S0305 were minimally altered, by less than twofold (Fig. 2B), but there was significant variation in virtually all genes examined for S0805 (Fig. 2E).

**Figure 3:**
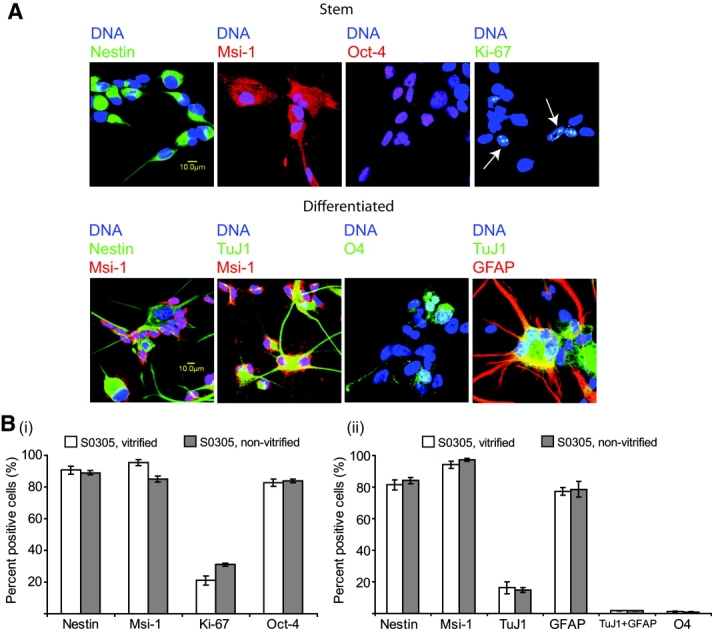
Vitrification preserves stemness and differentiation marker expression. **(A):** Immunofluorescent staining of representative sample S0305 with stem cell/precursor markers (Nestin, Msi-1, Oct-4, and Ki-67) (top panel) and multipotentiality markers (TuJ1, GFAP, and O4) (bottom panel). **(Bi):** Quantification of data in **(A)**, top panel. **(Bii):** Quantification of data in **(A)**, bottom panel. *p* > .05 for all samples pairs, indicating that there was no significant difference between vitrified and nonvitrified samples. Data for all other tumor neurosphere lines are available supporting information Figure 1A, 1B, and supporting information Table 2. Abbreviations: GFAP, glial fibrillary acidic protein; Msi-1, Musashi-1; TuJ1, neuron-specific class III beta tubulin.

We carried out immunofluorescent staining experiments to verify the stemness and multipotentiality profiles of vitrified and nonvitrified samples. All patients' tumor neurospheres demonstrated preservation of stem-like cells in vitrified and nonvitrified samples (Fig. 3A, top panel; Fig. 3Bi; supporting information Fig. 1A; supporting information Table 2A). As cell morphology changes accompany the induction of differentiation of neural stem cells, we assessed multipotentiality by scoring for neurons (TuJ1), astrocytes (GFAP), and oligodendrocytes (O4). All samples displayed the ability to differentiate into neurons, astrocytes, and oligodendrocytes (Fig. 3A, lower panel; Fig. 3Bii; supporting information Fig. 1B; supporting information Table 2B). In addition, we scored for differentiated cells staining positively for Nestin and Msi-1 stemness markers. We also scored for cells coexpressing GFAP and TuJ1. All patients' neurospheres, when differentiated, showed no significant differences between the vitrified and nonvitrified states, supporting the idea that vitrification preserves the multipotentiality property of the cells (supporting information Table 2B). We observed that all samples displayed 70%–95% Nestin- and Msi-1-stained cells despite being cultured under differentiating conditions, indicating the retention of self-renewal potential in otherwise normally terminally differentiated cells (Fig. 3A, lower panel; supporting information Table 2B). Work by several other investigators has also documented this phenotype [38,39]. This may in turn reflect an aberrant regulatory pathway in cancer stem cells. Differentiated cells were detected that costained for GFAP and TuJ1; notably, S0306 differentiated cells expressed the highest proportion of such cells (Fig. 3A, lower panel; supporting information Table 2B). Previous work by others has also demonstrated the coexistence of such normally distinct neural developmental pathways [14,38–40]; these pathways may characterize an abnormality in tumor-initiating cells.

### Vitrified Tumor Neurospheres Demonstrate Secondary Sphere Formation and Self-Renewal Potential

**Figure 4:**
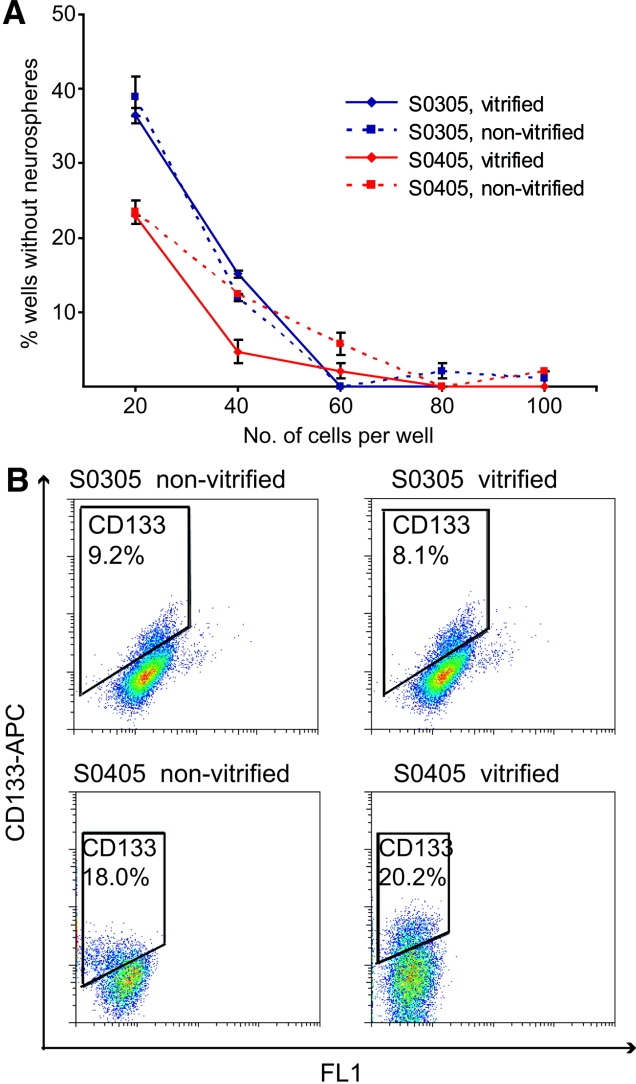
Tumor spheres possess self-renewal capability and maintain CD133 subpopulation. **(A):** Neurospheres were dissociated into single cells, plated at decreasing cell numbers, and analyzed for their ability to form secondary neurospheres. Representative plots are shown for two samples, S0305 and S0405. *p* > .05 for all sample pairs, indicating that vitrified and nonvitrified samples maintained self-renewal capability. Data for all other samples are shown in supporting information Figure 2A. **(B):** Tumor neurospheres were dissociated into single cells and stained with anti-CD133/2 antibody conjugated to APC according to the manufacturer's instructions (Miltenyi Biotec). CD133 percentage was determined by fluorescence-activated cell sorting. Each vitrified or nonvitrified sample was gated according to its own matched isotype control. Shown are representative plots of two patients' glioblastoma multiforme spheres, S0305 and S0405. Data for all other samples are shown in supporting information Figure 2B. Abbreviation: APC, allophycocyanin.

To investigate the stem cell frequency and self-renewal potential of our tumor neurospheres, we dissociated neurospheres into single cells, dispensed them into 96-well plates at decreasing cell numbers, and then scored them for secondary sphere formation after 7 days (Fig. 4A; supporting information Fig. 2A). Cell clustering played no apparent role in sphere formation, as cells were plated at clonal densities [14]. As neurospheres are heterogeneous and the neurosphere assay does not distinguish initially proliferating neural precursors from bona fide stem cells with self-renewal potential [27,28], we sought to carry out sequential minimal dilution assays for at least three passages, which confirmed that these single-cell-derived tumor spheres possessed the potential to grow infinitely, underscoring self-renewal as an important criterion for brain tumor-initiating cells [41]. The proportion of sphere-forming cells remained stable throughout the course of culture (>6 months), indicating asymmetrical cell divisions. There was no significant difference noted between the vitrified and nonvitrified samples of all patients' tumor neurosphere lines except for S0805, indicating that the vitrification procedure does not reduce the secondary sphere-forming ability of these cells (Fig. 4A; supporting information Fig. 2A). Moreover, the CD133-expressing population within the spheres that is often associated with tumor-initiating potential [10,42,43] was also maintained throughout the course of culture (>6 months; Fig. 4B; supporting information Fig. 2B).

### Vitrification Preserves the Karyotypic Hallmarks of Glioblastoma Multiforme

**Figure 5:**
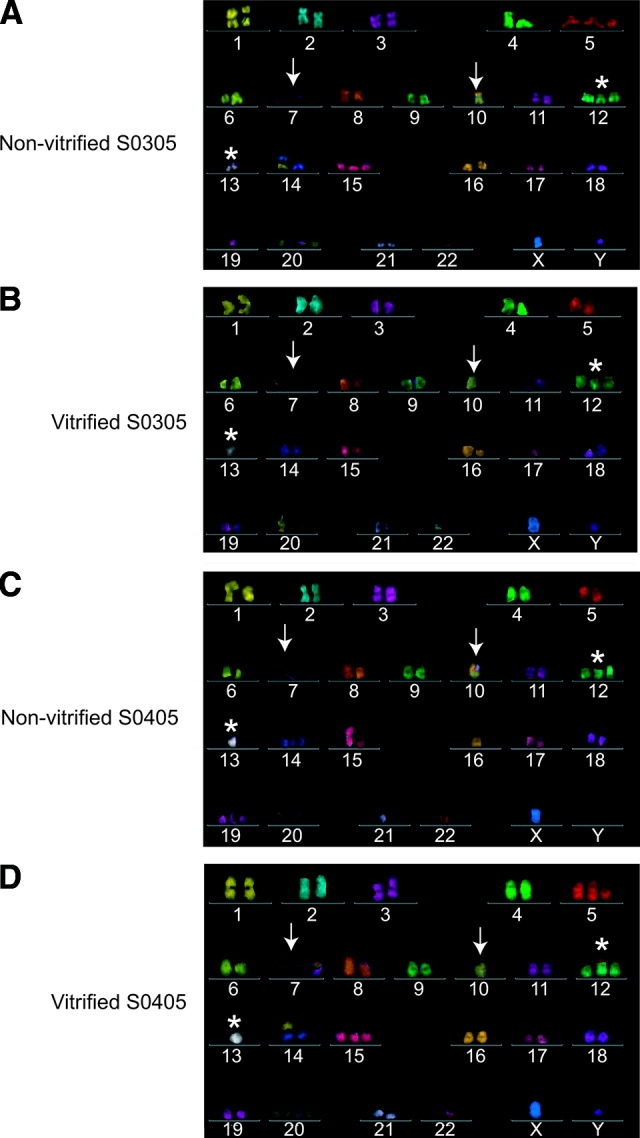
Vitrified neurospheres maintain karyotypic integrity and glioblastoma multiforme hallmarks. Single 2 × 10^5^ cells from dissociated neurospheres were karyotyped by metaphase-fluorescent in situ hybridization (mFISH) analyses according to the manufacturer's instructions (MetaSystems XCyte mFISH). Arrows point to polysomy of chromosome 7 and loss of chromosome 10. Asterisks indicate aneusomy of chromosomes 12 and 13. Data for all other neurosphere lines are shown in supporting information Figure 3.

To further demonstrate the tumor origin of our spheres, as well as to ascertain that vitrification itself preserves the karyotypic integrity and hallmarks of GBM, we karyotyped all patients' neurospheres before and after the vitrification process. Our data indicate that all spheres were of tumor origin and preserved their karyotypic integrity, as well as maintaining the hallmarks of GBM, in both vitrified and nonvitrified samples (Fig. 5; supporting information Fig. 3). Notably, polysomy of chromosome 7 and loss of chromosome 10 were present. This is consistent with a previous report by Singh et al. [10]. In addition, we observed aneusomy of chromosomes 12 and 13 across all five patients' tumor neurospheres. S0805 nonvitrified cells exhibited an altered karyotype compared with vitrified cells (supporting information Fig. 3C). As S0805 nonvitrified cells had been in vitro passaged for the longest period compared with all other lines (more than 50 passages), it is probable that this resulted in changes in proliferation rate, self-renewal potential, and gene expression, as previously shown.

### Tumor Neurospheres Re-Create Glioma Pathophysiology in NOD/SCID Mice

**Figure 6:**
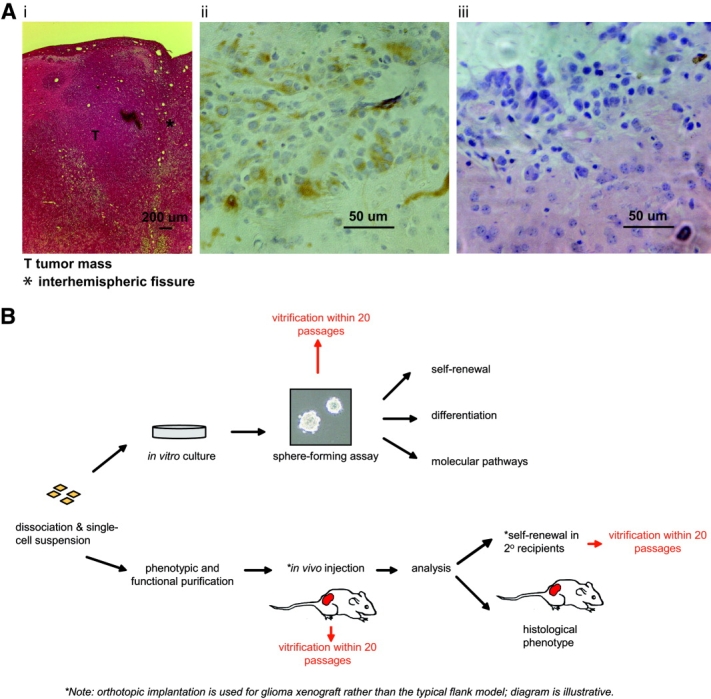
Vitrified glioblastoma multiforme spheres form tumor xenografts that recapitulate glioma pathophysiology. Two hundred thousand cells from dissociated tumor neurospheres were stereotaxically implanted into NOD/SCID mice. Animals were sacrificed by transcardiac perfusion upon presentation of neurological deficits. A representative tumor xenograft created from S0305 cells is shown. Tissue sections were stained with hematoxylin and eosin **(Ai)**. The presence of human cells in tumor xenografts was confirmed by staining with an anti-human vimentin antibody (clone V9; Zymed Laboratories) **(Aii)**. **(Ai)** represents the glioma mass formed (asterisk denotes interhemispheric fissure). Note the infiltrative tumor margin in **(Aiii)**. **(B):** A model is illustrated in which vitrification can be combined with in vivo serial passaging to maintain a repository of brain tumor-initiating cells. We propose that the vitrification method can be used at indicated stages (red) to cryopreserve tumor-initiating cells. These cells can subsequently be thawed and expanded at a later stage for biological assays, such as drug screening, or subjected to serial passaging in NOD/SCID mice. Vitrification permits long-term storage of such cells without changes to their genotypic and functional properties. Abbreviation: T, tumor mass.

The abilities to recapitulate tumor pathophysiology in an immune-compromised animal model and to serially transplant the tumor provide unequivocal evidence for the function of a cancer stem cell [41]. Importantly, when our vitrified tumor neurospheres grown under serum-free conditions were orthotopically implanted in NOD/SCID mice, tumor xenografts formed that recapitulated glioma pathophysiology. The intracranial tumors of S0305 and S0405 demonstrated extensive infiltration into the surrounding cerebral cortex, a pathognomonic feature of human GBMs [10,14] (Fig. 6A). Tumors formed by S0805 and S0807 were smaller in size due to an earlier period of sacrifice but nevertheless demonstrated similar infiltrative margins [22]. S0306 cells formed a tumor that exhibited a well-circumscribed margin (data not shown). These tumors were also serially transplantable in secondary recipients for two of the lines tested (two of three mice; S0305 and S0405), conclusively demonstrating the in vivo self-renewal potential of GBM-derived tumor-initiating cells. In contrast, mice implanted with serum-grown cells generated spatially constrained gliomas with clear delineated margins, nonreflective of actual glioma growth patterns (data not shown) [15]. This underscores the importance of using tumor neurospheres grown in serum-free conditions as the relevant model for further investigations.

### Gene Expression Studies Demonstrate the Clustering of Vitrified and Nonvitrified Samples and Distinguish Tumor Spheres from Their Differentiated Forms and Primary Tumors

**Figure 7:**
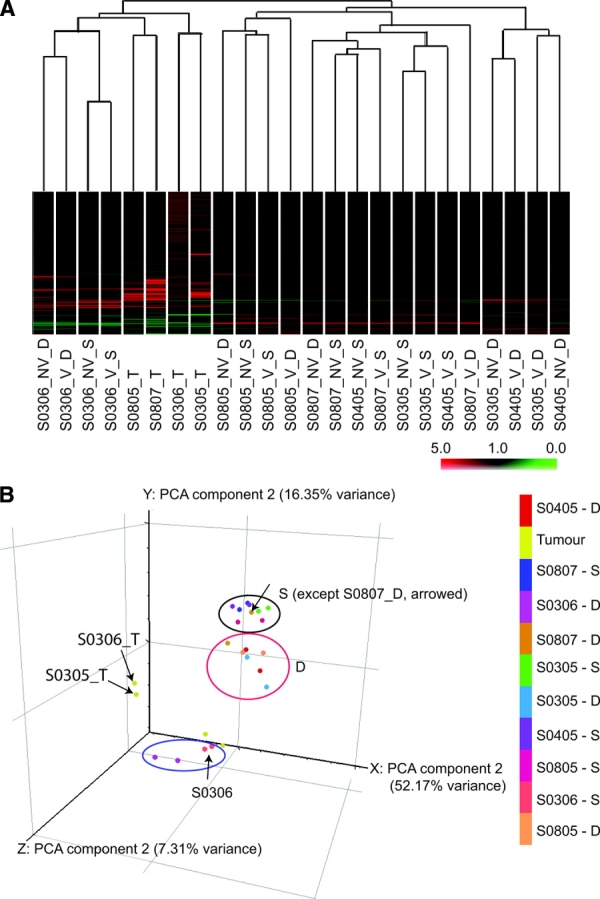
Vitrification preserves genetic profiles of glioblastoma multiforme spheres, which are genetically distinct from primary tumors and differentiated cells. **(A):** Dendrogram determined by unsupervised hierarchical cluster analysis of all patients' tumor neurospheres (V and NV) cultivated under serum-free conditions supplemented with growth factors (S-suffix) or differentiated by withdrawal of growth factors with addition of serum (D-suffix). Samples with the T-suffix represent the original primary tumor specimen. Numbers represent codes assigned to specimens. **(B):** PCA of all samples in **(A)** showing the distinct genetic profiles of S, D, and T clusters. S0306 tumor spheres associated with the T cluster. Abbreviations: D, differentiated; NV, nonvitrified; PCA, principal component analysis; S, serum-free; T, tumor; V, vitrified.

If vitrification is to be an efficient method for cryopreserving tumor-initiating cells, then we expect that vitrified and nonvitrified samples should generate transcriptome profiles that cluster together, indicating the genetic stability of the samples. We thus performed microarray gene expression analyses on all five patients' tumor neurosphere lines (vitrified and nonvitrified), as well as on their differentiated progenies (vitrified and nonvitrified) and primary tumor specimens. Indeed, unsupervised cluster analysis showed that the vitrified form of each sample in either the stem/progenitor or differentiated state clustered together with its respective nonvitrified form (Fig. 7A, 7B). This supports our finding that vitrification preserves the genetic profile of brain tumor-initiating cells. An exception was S0807; the transcriptome profile of the differentiated form resembled that of the S cluster. Upon induction of differentiation, S0807 cells tended to grow in compact patches compared with all other samples (unpublished observations); thus, it is plausible that they remained relatively undifferentiated, resulting in a transcriptome that resembled that of the stem-like cells. In addition, tumor neurospheres consisting of stem and progenitor cells were also genetically distinct from their differentiated forms and primary tumors. S0306 undifferentiated spheres however clustered with the primary tumors. We believe this reflects a different phenotypic and genotypic subtype of GBM neurospheres [22,44].

## DISCUSSION

Tissue repositories have traditionally been maintained either as frozen samples stored in liquid nitrogen tanks, or embedded in paraffin wax. Although both methods of storage allow the retrieval of cellular material, it does not allow the isolation and subsequent cultivation of live cells from the stored tumor. In this study, we present data for the first time on a modified vitrification method for human brain tumor neurospheres that enriches for tumor-initiating cells, more commonly referred to as cancer stem cells. Such a method evaluates essential stem cell-like properties, multipotentiality capacity, genotypic profile, and ability to recapitulate glioma pathophysiology. Vitrification now provides a solution to the long-term storage of tumor-initiating cells without the need to maintain a constant supply of immune-compromised animals of suitable ages to in vivo serially passage the cells. With the vitrification approach, a glass-like solidification of the freezing solution is achieved by using a high concentration of cryoprotectant and rapid cooling. This method eliminates cell injury due to ice crystal formation. Although various cryopreservation techniques have been developed for a range of cells, such as human/mouse embryonic stem cells [18,45] and mouse neural precursor cells [46–48], these studies have largely relied on gross morphological appearances and have ignored examining the genetic profiles and quantitative analysis of cell types (both stem and differentiated forms) of samples. For validation of vitrification as a method of cryopreservation for brain tumor-initiating cells, the cellular heterogeneity of tumor cells and their ability to recapitulate glioma pathophysiology would have to be taken into consideration.

Standard freezing techniques with high serum content have been used in many cellular systems because of their less complex preparatory steps. Previous work has evaluated the use of such a method in the cryopreservation of human embryonic stem cells, which resulted in differentiated outgrowths [45]. Here, we demonstrate that although freezing with 90% FBS yielded the best viability of tumor neurospheres, it also resulted in differentiated outgrowths. Serum contains many unknown growth factors or cytokines that can induce differentiation of stem cells when applied at high concentrations [45,49]. Nevertheless, given the significantly better viability, slow freezing with high serum presents an attractive alternative that should be explored in future studies. We show that vitrification maintains essential stem/progenitor-like properties, multipotentiality, and genotypic profiles. Importantly, the vitrified cells retain the capacity to form tumor xenografts that recapitulate glioma pathophysiology. To conclusively demonstrate the stemness of GBM-derived tumor-initiating cells, we performed sequential transplantation experiments. This was done in analogy with the classic hematopoietic serial repopulation paradigm used to identify true hematopoietic stem cells [50]. The successful, sequential generation of brain tumors provides evidence that we have isolated and maintained bona fide tumor-initiating cells [41].

Our microarray gene expression data indicate the clustering of vitrified and nonvitrified samples in each tumor specimen, consistent with this freezing technique maintaining the genetic profiles of the tumor neurospheres. To our surprise, the S0805 nonvitrified sample, which we previously concluded to have undergone changes due to prolonged in vitro expansion, now clustered with its vitrified form. Lee et al. [15] demonstrated the genetic stability of tumor neurosphere lines growing in culture for more than 70 passages by examining their transcriptome profiles. We believe that although transcriptome profiles reflect overall gene transcript changes, one needs to exercise caution in extrapolating the data to indicate stability of cell lines. Importantly, parallel analyses of a combination of essential stem cell characteristics should be performed to evaluate the quality of a tumor neurosphere line.

We observed that S0306 spheres generated a profile distinct from all other tumor sphere samples and instead associated more closely with primary tumors. Interestingly, S0306 spheres displayed semiadherent growth patterns compared with all other free-floating spheres. This is consistent with the presence of tumor sphere subtypes as previously reported [22,44]. Günther et al. [22] was able to ascertain that the gene expression profile of the semiadherent spheres contained genes more commonly expressed in GBM tumor tissue specimens. This is in contradistinction with the profiles of free-floating spheres. Indeed, the genetic profile of S0306 spheres was associated more closely with that of primary tumors (Fig. 7B).

We highlight that although primary tumors containing a mix of different cell types can display transcriptome profiles that associate closely with each other (Fig. 7B, S0306T and S0305T), the genetic profiles of their presumed cell of origin (S0306-S and S0305-S) can be significantly different. This may reflect a limitation in evaluating a heterogeneous tumor mass as opposed to a population enriched for tumor-initiating cells. In gene expression studies, Lee et al. [15] reported that tumor spheres clustered together with parental tumor samples, and this clustering differed as matched samples were differentiated in serum-containing media. They inferred that because tumor spheres contained gene expression and biology similar to those of parental tumors, the tumor spheres therefore represented a more reliable model system compared with serum-grown cancer cells. We believe that in the cluster analysis, the tumor spheres clustering together with the parental tumors would depend on the abundance of tumor stem/progenitor cells in the parental tumor. If cancer stem-like cells form only a minority of the parental tumor, this clustering would not be evident. Indeed, work by Clement et al. [51] showed the stemness signature associating more closely with grade III astrocytomas than with grade IV GBM tumors. In our study, our parental tumors clustered more closely with the differentiated forms, likely indicating that the parental tumors that we collected contained more differentiated cells. Importantly, we have shown that our tumor spheres were able to reform tumor xenografts in NOD/SCID mice that recapitulated glioma pathophysiology (Fig. 6A), thus confirming their identity as tumor-initiating cells [14]. These spheres were also more resistant to the effects of temozolomide (a commonly used clinical drug) compared with serum-grown glioma cells (supporting information Fig. 5), thereby underscoring the significance of focusing on this cellular subpopulation.

## CONCLUSION

Our data illustrate for the first time that tumor stem-like cells are genetically distinct from their differentiated forms, which in turn associate more closely with the parental tumors. This highlights the significance of designing therapeutic agents targeted at the relevant minority tumor-initiating population in addition to targeting the other majority more differentiated tumor mass. Prolonged in vitro culturing of S0805 tumor neurospheres resulted in karyotypic, genotypic, and phenotypic changes, further highlighting the importance of our vitrification technique in cryopreserving such cells at early passages of primary culture establishment. Our method may be applicable to other neoplastic stem-like cells grown in a spheroid manner, such as those isolated from the breast (mammospheres) [52,53], prostate (prostaspheres) [54,55], head and neck [56], and lung [57] cancers. These spheres have been shown to be of clonal origin and to derive from primitive cells. More importantly, they can be dissociated and passaged for more than 10 generations, suggesting the existence of a population of cells with extensive self-renewal capacity. Our work therefore supports the use of the vitrification procedure in establishing a brain tumor-initiating cell repository for facilitating further investigative work focused on this cellular subpopulation, and possibly for future drug targets. We envisage that a combination of vitrification and in vivo serial passaging in NOD/SCID mice will provide a convenient means of preserving the tumor-initiating population (Fig. 6B).
