# Supplementary material for: Cryopreservation of Neurospheres Derived from Human Glioblastoma Multiforme
Source: Stem Cells. 2009 Jan;27(1):29–39. doi: 10.1634/stemcells.2008-0009 (PMC2729678; doi:10.1634/stemcells.2008-0009)
Supplement: Supplementary file 5 [file stem0027-0029-SD5.pdf]

Supplemental Table 1 Tang et al.

Primers used for real-time PCR

mRNA target	Oligonucleotides (5’→ 3’)	Product Size (bp) / Temperature(°C)
Bmi-1	Forward: AAA GAT ACT TAC GAT GCC CAG<br>Reverse: GAA GTG GAC CAT TCC TTC TC	183<br>55
CD133	Forward: GAA CAA GTT TAC AGT GAC TGC<br>Reverse: TGC GTT GAA GTA TCTT TGA CG	444<br>55
GAPDH	Forward: GGA AGG TGA AGG TCG GAG TC<br>Reverse: GTC TTG TGG GTG GCA GTG AT	556<br>55
GFAP	Forward: GGG ATG GAG AGG TCA TTA AGG<br>Reverse: GGG TGA GTT TCT TGT TAG TTG G	287<br>55
MOBP	Forward: GAC TCA TTG CTT CAC AAC CC<br>Reverse: CTT CAA AGT ACT CCA GGC AG	152<br>55
Musashi-1 (Msi-1)	Forward: GTT TCG GCT TCG TCA CTT TC<br>Reverse: GAG TCA CCA TCT TGG GCT GT	135<br>55
Nanog	Forward: AGC TAC AAA CAG GTG AAG AC<br>Reverse: CTC CAG GTT GAA TTG TTC CA	218<br>55
Nestin	Forward: AGA CAC CTG TGC CAG CCT TTC<br>Reverse: CTG CTG CAA GCT GCT TAC CAC	469<br>55
OCT-4	Forward: GGT TCT ATT TGG GAA GGT ATT CAG<br>Reverse: GGT TTC TGC TTT GCA TAT CTC	119<br>55
Sox-2	Forward: AGC TGG GAT AGG CCT CAC TT<br>Reverse: TGA ATC CAT TTC GGC TTT TC	187<br>55
β-Tubulin III (TuJ1)	Forward: CTT CAT TTC CCG TCA GTG TG<br>Reverse: TAT AAT CCT GTC TGG GTA CTC CT	225<br>55
