# Supplementary material for: Cryopreservation of Neurospheres Derived from Human Glioblastoma Multiforme
Source: Stem Cells. 2009 Jan;27(1):29–39. doi: 10.1634/stemcells.2008-0009 (PMC2729678; doi:10.1634/stemcells.2008-0009)
Supplement: Supplementary file 6 [file stem0027-0029-SD6.pdf]

Supplemental Table 2 Tang et al.

A. Immunofluorescence quantification (% mean ± SEM) of undifferentiated primary cell lines

Primary GBM	Markers							
	Nestin		Msi-1		Ki-67		Oct-4	
	V	NV	V	NV	V	NV	V	NV
S0305	90.7 ± 2.6	89.0 ± 1.5	95.4 ± 2.0	84.9 ± 2.0	21.0 ± 2.8	31.0 ± 0.8	82.6 ± 2.3	83.9 ± 1.2
S0405	97.0 ± 0.7	94.7 ± 0.2	95.0 ± 0.2	96.1 ± 1.4	19.7 ± 0.3	21.6 ± 1.8	73.5 ± 5.7	74.5 ± 5.0
S0807	99.2 ± 0.6	96.4 ± 0.8	98.0 ± 0.1	98.5 ± 0.7	21.3 ± 1.8	22.0 ± 3.8	93.8 ± 1.5	95.7 ± 0.2
S0306	97.6 ± 0.6	93.4 ± 1.4	83.2 ± 2.5	87.5 ± 0.6	34.3 ± 3.9	43.6 ± 4.3	86.3 ± 4.3	90.8 ± 2.7
S0805	94.0 ± 2.7	92.4 ± 2.2	92.7 ± 0.7	97.6 ± 0.6	24.3 ± 0.5	32.8 ± 5.3	91.4 ± 3.5	95.1 ± 0.3

B. Immunofluorescence quantification (% mean ± SEM) of differentiated primary cell lines

Primary GBM	Markers											
	Nestin		Msi-1		TuJ1		GFAP		TuJ1+GFAP		O4	
	V	NV	V	NV	V	NV	V	NV	V	NV	V	NV
S0305	81.5 ± 3.2	84.1 ± 2.0	94.1 ± 2.2	97.2 ± 1.0	16.2 ± 3.8	14.7 ± 1.5	77.3 ± 2.4	78.6 ± 4.9	2.0 ± 0.2	2.0 ± 0.2	1.2 ± 0.3	1.0 ± 0.3
S0405	71.4 ± 4.3	71.3 ± 2.6	92.0 ± 0.7	91.2 ± 2.6	25.5 ± 3.9	25.2 ± 1.6	53.9 ± 1.8	50.1 ± 6.7	6.4 ± 1.5	6.4 ± 1.2	0.0 ± 0.0	0.0 ± 0.0
S0807	93.1 ± 1.0	91.5 ± 2.9	98.2 ± 1.0	97.7 ± 0.4	35.8 ± 1.8	38.4 ± 3.1	49.4 ± 4.8	50.9 ± 5.3	8.0 ± 1.0	8.0 ± 1.4	6.3 ± 3.5	4.9 ± 0.8
S0306	95.5 ± 0.4	95.9 ± 2.9	97.2 ± 1.2	100.0 ± 0.0	86.5 ± 5.5	95.0 ± 2.0	92.4 ± 3.0	97.9 ± 2.2	77.0 ± 7.5	85.0 ± 9.0	0.0 ± 0.0	0.0 ± 0.0
S0805	85.7 ± 1.1	84.8 ± 3.7	97.1 ± 0.5	98.3 ± 1.2	15.5 ± 2.7	13.7 ± 1.1	85.4 ± 2.5	92.3 ± 1.7	4.7 ± 0.6	6.7 ± 0.4	20.9 ± 2.0	17.0 ± 0.9
